# Preoperative Non-Invasive Prediction of Breast Cancer Molecular Subtypes With a Deep Convolutional Neural Network on Ultrasound Images

**DOI:** 10.3389/fonc.2022.848790

**Published:** 2022-07-18

**Authors:** Chunxiao Li, Haibo Huang, Ying Chen, Sihui Shao, Jing Chen, Rong Wu, Qi Zhang

**Affiliations:** ^1^ Department of Ultrasound, Shanghai General Hospital, Shanghai Jiao Tong University School of Medicine, Shanghai, China; ^2^ The SMART (Smart Medicine and AI-Based Radiology Technology) Lab, School of Communication and Information Engineering, Shanghai University, Shanghai, China

**Keywords:** deep convolutional neural network, ultrasound, breast cancer, molecular subtype, luminal A, triple-negative breast cancer

## Abstract

**Purpose:**

This study aimed to develop a deep convolutional neural network (DCNN) model to classify molecular subtypes of breast cancer from ultrasound (US) images together with clinical information.

**Methods:**

A total of 1,012 breast cancer patients with 2,284 US images (center 1) were collected as the main cohort for training and internal testing. Another cohort of 117 breast cancer cases with 153 US images (center 2) was used as the external testing cohort. Patients were grouped according to thresholds of nodule sizes of 20 mm and age of 50 years. The DCNN models were constructed based on US images and the clinical information to predict the molecular subtypes of breast cancer. A Breast Imaging-Reporting and Data System (BI-RADS) lexicon model was built on the same data based on morphological and clinical description parameters for diagnostic performance comparison. The diagnostic performance was assessed through the accuracy, sensitivity, specificity, Youden’s index (YI), and area under the receiver operating characteristic curve (AUC).

**Results:**

Our DCNN model achieved better diagnostic performance than the BI-RADS lexicon model in differentiating molecular subtypes of breast cancer in both the main cohort and external testing cohort (all p < 0.001). In the main cohort, when classifying luminal A from non-luminal A subtypes, our model obtained an AUC of 0.776 (95% CI, 0.649–0.885) for patients older than 50 years and 0.818 (95% CI, 0.726–0.902) for those with tumor sizes ≤20 mm. For young patients ≤50 years, the AUC value of our model for detecting triple-negative breast cancer was 0.712 (95% CI, 0.538–0.874). In the external testing cohort, when classifying luminal A from non-luminal A subtypes for patients older than 50 years, our DCNN model achieved an AUC of 0.686 (95% CI, 0.567–0.806).

**Conclusions:**

We employed a DCNN model to predict the molecular subtypes of breast cancer based on US images. Our model can be valuable depending on the patient’s age and nodule sizes.

## Introduction

Due to the development of tumor biology, genomics, bioinformatics, and other areas of basic research, the treatment of breast cancer has transformed from the traditional surgery mode into a precision medicine mode (1). Clinically, based on the state of hormone receptors (estrogen receptor [ER] and progesterone receptor [PR]), human epidermal growth factor receptor-2 (HER2), and Ki67 proliferation index, breast cancer is categorized into different molecular subtypes that have distinct risk profiles and treatment schemas (2, 3). Four breast cancer molecular subtypes have been described, namely, luminal A (hormone receptor positive, HER2 negative), luminal B (hormone receptor positive, HER2 positive), HER2-enriched (hormone receptor negative, HER2 positive), and triple-negative (hormone receptor negative, HER2 negative) subtypes. This is the basis of precision medicine for breast cancer. One study from *JAMA* demonstrated that 63% of the overall mortality reduction of breast cancer was associated with treatments of chemotherapy, hormone therapy, and trastuzumab, and the associations varied by breast cancer molecular subtypes (4). The classification of molecular subtypes depends on postoperative pathology, which is significantly delayed for the rational preoperative treatment formulation. Therefore, non-invasive methods for molecular subtypes of breast cancer before surgery are crucial for further arrangement and have become the focus of current research.

In order to align with the concept of precision medicine, many attempts have been taken by medical imaging to provide a promising solution for preoperative non-invasive prediction of breast cancer molecular subtypes. As a routine modality for breast diseases, there have been recent studies of ultrasound (US) on molecular typing of breast masses by morphological features (5–7). However, because of the operator dependence and instability of US images, the claimed relevant sonographic features such as margins and posterior acoustic features might be indefinite and unreliable. Therefore, in the actual clinical work, it is almost impossible for radiologists to predict the molecular subtypes of breast masses by only relying on visual observation of US images. Additional diagnostic assistance is urgently needed for real-world radiologists to cope with this challenge.

At present, several studies combined medical images with machine learning or deep learning (DL) approaches, which are more accurate and convenient to predict molecular subtypes preoperatively based on US, mammography, and MRI images (8–11). Different from the hand-crafted feature extraction of machine learning methods, DL has been proved to be more labor-saving and superior by automatically extracting features from raw data (12). The deep convolutional neural network (DCNN) consisting of consecutive layers for feature extraction is one of the most popular architectures in the DL family nowadays (13).

Based on pathophysiological and epidemiological results, triple-negative breast cancer occurs more frequently in women who are younger, and the tumors are usually larger in size. Furthermore, in China, the peak ages of breast cancer are in the 40–50-year age group (14, 15). As a whole, patient age and lesion size have a certain reference value for the accurate diagnosis of molecular subtypes. Hence, firstly, the purpose of this study was to predict the molecular subtypes of breast cancer through DCNN architecture based on primary breast US images. Furthermore, we explored the diagnostic efficacy in different age groups (≤50 and >50 years) and tumor size groups (≤20 and >20 mm).

## Materials and Methods

### Patients and Data

**Figure 1:**
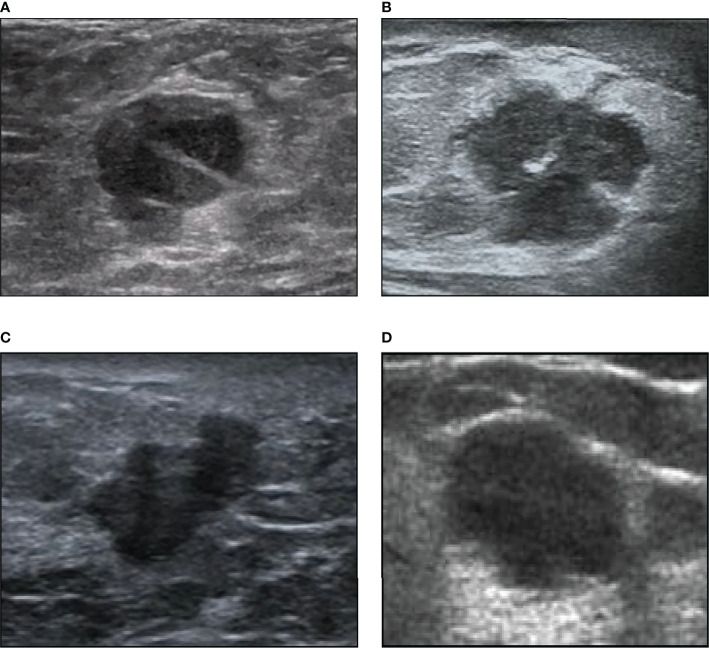
Examples of our ultrasound image dataset. **(A)** A 72-year-old woman with intraductal papillary breast carcinoma (1.6 cm) of luminal A subtype. **(B)** A 56-year-old woman with invasive ductal breast carcinoma (1.7 cm) of luminal B subtype. **(C)** A 40-year-old woman with invasive micropapillary breast carcinoma (1.5 cm) of HER2+ subtype. **(D)** A 39-year-old woman with invasive ductal breast carcinoma (1.9 cm) of triple-negative subtype.

The datasets used in this study were collected from two hospitals, one as the main cohort for training and internal testing collected between January 2008 and August 2019 and the other as the external testing cohort collected between June 2021 and February 2022. This retrospective study was approved by the Institutional Review Board of our hospital (No. 2019KY055). Informed consent was waived by the board. The inclusion criteria of this study were as follows: 1) all the breast masses were pathologically proven, 2) patients with complete information of US examinations, 3) the patients had not undergone other treatments, and 4) complete immunohistochemical (IHC) marker (ER, PR, HER2, and Ki-67) result on histopathology. The exclusion criteria were as follows: 1) US images with poor quality and 2) the pathological results and IHC markers were incomplete for molecular subtyping. ER and PR positivity was defined as the detection of ≥1% positive staining in tumor nuclei. HER2 status was graded as 0, 1+, 2+, or 3+. A score of 3+ staining was defined as HER2 positive, and scores of 0 and 1+ were considered as HER2 negative. As for 2+ staining, HER2 gene amplification by fluorescence *in situ* hybridization (FISH) should be taken for final diagnosis. Ki-67 expression was graded as low (<14%) or high (≥14%). Breast cancers were categorized into four subtypes on the basis of their receptor status. The subgroups defined were as follows: luminal A (ER and/or PR+, HER2−), luminal B (ER and/or PR+, HER2+), HER2-positive (ER and PR−, HER2+), and triple-negative (ER−, PR−, and HER2−) (**Figure 1**). Finally, a total of 1,012 breast cancer lesions (20.85 ± 11.33 mm; range, 7.8–104 mm) from 1,012 patients (57.14 ± 13.09 years; range, 25–90 years) with 2,284 US images were involved as the main cohort (center 1), including 248 cases (548 images) of luminal A subtype, 457 cases (1077 images) of luminal B subtype, 105 cases (222 images) of HER2+ subtype, and 202 cases (437 images) of triple-negative subtype. Another cohort of 117 breast cancer lesions (22.79 ± 12.06 mm; range, 4–65 mm) from 117 patients (59.25 ± 11.91 years; range, 33–85 years) with 153 US images (center 2) was used as the external testing cohort, including 41 cases (52 images) of luminal A subtype, 47 cases (58 images) of luminal B subtype, 13 cases (20 images) of HER2+ subtype, and 16 cases (23 images) of triple-negative subtype.

The US images in this study were acquired by several different US equipment in our medical center, including Philips (IU22; Amsterdam, the Netherlands), Aixplorer (Super Imagine; Aix-en-Provence, France), GE Healthcare (LOGIQ E9; Pittsburgh, PA, USA), Hitachi (EUB 8500; Tokyo, Japan), Esaote (MyLab™Twice; Genoa, Italy), and Siemens (Sequia512 and ACUSON S3000; Munich, Germany) with linear transducers of frequency 5–12 MHz. Moreover, clinical data on patient age, US tumor size (short diameter and long diameter), and aspect ratio were also collected for feature extraction.

### Image Annotation and Dataset Partition

The labeling tool of LabelImg (https://github.com/tzutalin/labelImg) was used to crop the regions of interest (ROIs) from the original breast US images with rectangular boxes. The annotation was manually processed and confirmed by a breast radiologist with over 5 years of experience in breast US interpretation.

The main cohort dataset was split into two parts, a training set and a test set, according to the patient count at a ratio of 4:1. The image numbers of each subset in the main cohort are shown in **Table 1**.

**Table 1:** The main cohort dataset with different groups of tumor sizes and patient ages.

Tumor size groups (mm)	Images (cases)		Total
	≤20	>20	
Luminal A	401 (189)	147 (59)	548 (248)
Luminal B	542 (246)	535 (211)	1,077 (457)
HER2+	112 (51)	110 (54)	222 (105)
Triple-negative	197 (94)	240 (108)	437 (202)
Age groups (years)	≤50	>50	
Luminal A	148 (60)	300 (188)	548 (248)
Luminal B	485 (182)	592 (275)	1,077 (457)
HER2+	75 (34)	147 (71)	222 (105)
Triple-negative	114 (53)	323 (149)	437 (202)
Total			2,284 (1,012)

### Deep Convolutional Neural Network Model

In this paper, we proposed a DCNN model, which combined the B-mode US image of breast cancer and clinical information to classify the molecular subtypes of breast cancer. We used the pre-trained convolutional neural networks on the ImageNet dataset to extract the high dimensional features of B-mode US images. The clinical information was encoded through a neural network and finally became a 512-dimensional feature vector. Then both the features of B-mode US images and the feature vector of clinical information were concatenated for classification by a softmax algorithm.

We fine-tuned three different DCNN models on our B-mode US image dataset, including EfficientNet, DenseNet-121, and VGGNet-16. Before the images were input into the network, they were adjusted to a size of 224 × 224 pixels and normalized. In order to solve the problem of overfitting, data augmentation including flipping, rotation, cropping, and contrast transformation was used during the training of networks.

**Figure 2:**
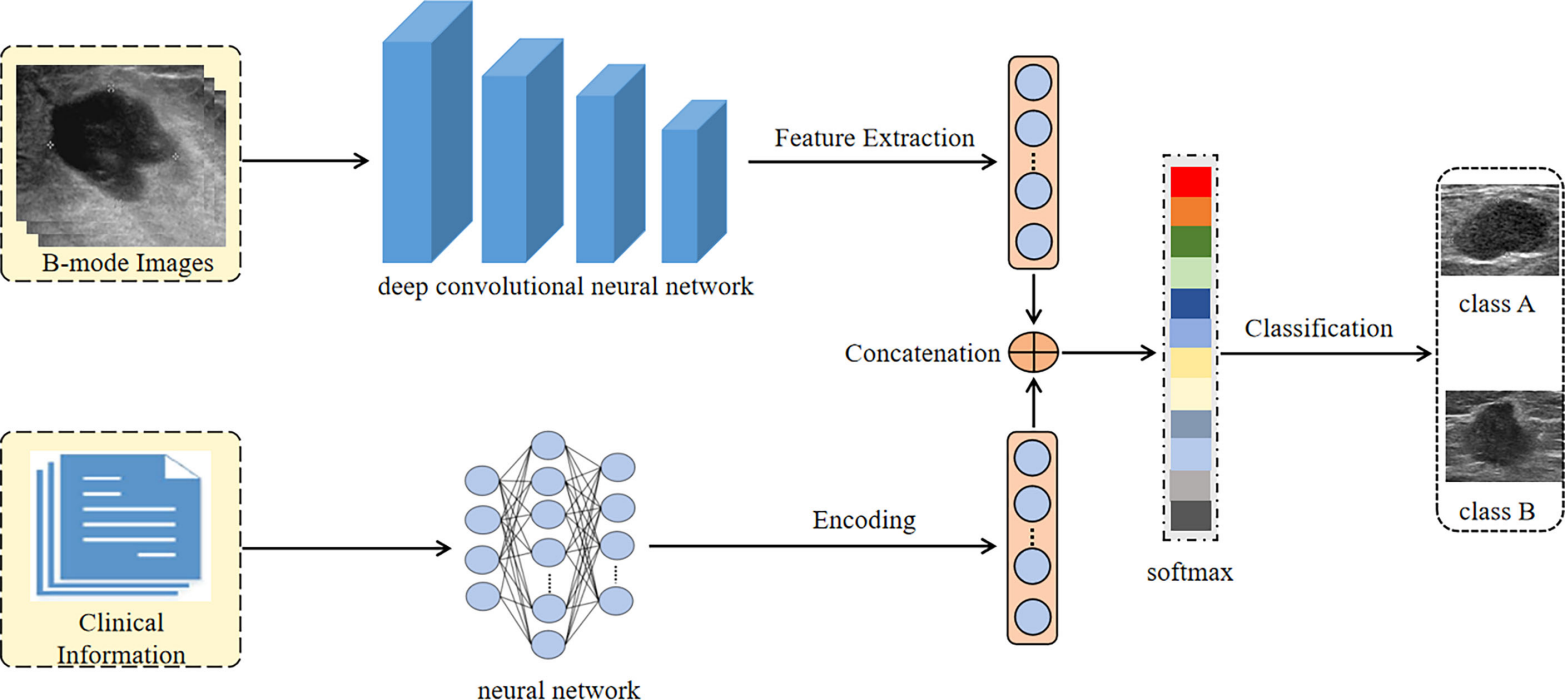
The processing flow of our deep convolutional neural network architecture.

We trained the DCNN with the stochastic gradient descent (SGD) optimizer accomplished in a computer with two NVIDIA 2080Ti graphic processing units (GPUs) and 256 GB of random access memory. To solve the problem of unbalanced samples, the focal loss strategy was used. The batch size, learning rate, and maximum iterations were set to 16, 0.005, and 100, respectively. The processing flow of our DCNN Architecture is shown in **Figure 2**.

### Breast Imaging-Reporting and Data System Lexicon Model

Morphological characteristics of each mass were classified by two radiologists who were blinded to the clinical and pathological results of the breast lesions. If there was no initial agreement, the final result will be reached after discussion. The two radiologists had 7 and 20 years of experience in breast US interpretation.

In accordance with the American College of Radiology Breast Imaging Reporting and Data System (BI-RADS) lexicon, the morphological description included the following parameters: the shape (oval, round, or irregular), orientation (parallel or not parallel), margin (circumscribed, indistinct, angular, microlobulated, or spiculated), echo pattern (anechoic, hyperechoic, complex, hypoechoic, isoechoic, or heterogeneous), posterior acoustic features (no posterior acoustic features, enhancement, shadowing, or combined pattern), surrounding tissue (adjacent ducts changes, tissue edema, architectural distortion, skin thickening, skin retraction, and irregularity), calcification (outside a mass, in a mass, intraductal, or none), and vascularity distribution (none, in a mass, next to a mass, or in the surrounding tissues). These BI-RADS parameters were included as variables for BI-RADS lexicon modeling. Other included parameters were age, lesion number, lesion location, tumor size, and lymph node status in US (normal or abnormal).

Then we constructed a BI-RADS lexicon model based on the above parameters. The solver, penalty, and maximum iterations of the model were all adjusted for the best result during the training period.

### Statistical Analysis

The diagnosis performance was assessed according to the classification accuracy (ACC), sensitivity (SEN), specificity (SPC), Youden’s index (YI), and area under the receiver operating characteristic curve (AUC). The diagnosis performance was evaluated based on the patient level, not the image level. The bootstrapping analysis was performed on the AUC values of each group of experiments to obtain the 95% CIs. The paired t-test was applied to AUC values to measure the performance differences. Statistical analysis was computed using the Python (3.6) programming language and R Studio software (3.5.2). p < 0.05 was regarded as statistically significant.

## Results

### Deep Convolutional Neural Network Model vs. Breast Imaging-Reporting and Data System Lexicon Model

As shown in **Tables 2** and **3**, our DCNN model showed better performance compared with the BI-RADS lexicon model in all three experiments in terms of AUC values in both the main cohort and external testing cohort (all p < 0.001), which proved the validity of our model. In the main cohort, for distinguishing luminal A from non-luminal A subtypes, our DCNN model (DenseNet-121) achieved the highest AUC (0.717, 95% CI, 0.622–0.809) and ACC (80.1%) as compared with the BI-RADS lexicon model (0.628, 95% CI, 0.539–0.719 and 52.2%). The diagnostic SEN, SPC, and YI were 87.8%, 53.3%, and 41.1%, respectively. Our DCNN models of EfficientNet-B2 obtained a higher AUC of 0.601 (95% CI, 0.495–0.683) for differentiating the luminal from non-luminal subtypes compared with the BI-RADS lexicon model. Our DCNN models also achieved an AUC of 0.577 (95% CI, 0.455–0.698) and an ACC of 76.9% for differentiating the triple-negative (TN) and non-triple-negative (non-TN) subtypes, which was better than the BI-RADS lexicon model. In the external testing cohort, our DCNN model achieved better AUC values of 0.680 (95% CI, 0.579–0.780), 0.639 (95% CI, 0.518–0.760), and 0.560 (95% CI, 0.399–0.721) as compared with the BI-RADS lexicon model (0.622, 95% CI, 0.516–0.728; 0.462, 95% CI, 0.342–0.583; 0.433, 95% CI, 0.266–0.599) in all three experiments.

**Table 2:** Diagnostic performance of deep convolutional neural network model for differentiating breast cancer subtypes in the main cohort.

Experiment	Model	AUC	ACC (%)	SEN (%)	SPC (%)	YI (%)
Luminal A vs. non-luminal A	EfficientNet-B0	0.686	78.1	86.5	48.9	35.4
	DenseNet-121	0.717	80.1	87.8	53.3	41.1
	VGGNet-19	0.664	74.6	82.1	48.9	31.0
Luminal vs. non-luminal	EfficientNet-B2	0.601	64.2	53.7	68.4	22.1
	DenseNet-121	0.587	61.1	64.8	59.6	24.4
	VGGNet-19	0.561	64.2	48.1	70.6	18.7
Triple-negative vs. non-triple-negative	EfficientNet-B2	0.577	76.9	33.3	86.3	19.6
	DenseNet-121	0.565	58.1	60.6	57.5	18.1
	VGGNet-19	0.572	50.5	69.7	46.4	16.1

AUC, area under the curve; ACC, accuracy; SEN, sensitivity; SPC, specificity; YI, Youden’s index.

**Table 3:** Diagnostic performance of BI-RADS lexicon model for differentiating breast cancer subtypes in the main cohort.

Experiment	AUC	ACC (%)	SEN (%)	SPC (%)	YI (%)
Luminal A vs. non-luminal A	0.628	0.522	0.436	0.822	0.258
Luminal vs. non-luminal	0.494	0.668	0.222	0.846	0.068
Triple-negative vs. non-triple-negative	0.553	0.575	0.576	0.575	0.151

BI-RADS, Breast Imaging-Reporting and Data System; AUC, area under the curve; ACC, accuracy; SEN, sensitivity; SPC, specificity; YI, Youden’s index.

### The Effect of Age on Differentiating Molecular Subtypes

**Figure 3:**
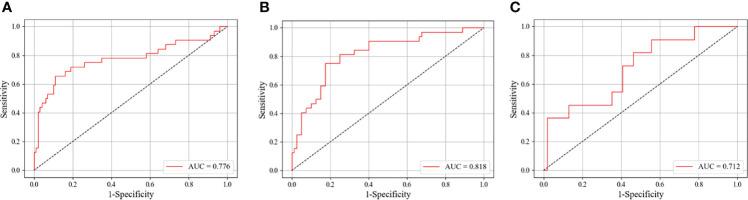
The ROC curves of our DCNN model in identifying different breast cancer molecular subtypes on the test set. **(A)** Classifying luminal A from non-luminal A subtypes among patients older than 50 years. **(B)** Classifying luminal A from non-luminal A subtypes for tumor sizes ≤20 mm. **(C)** Classifying triple-negative from non-triple-negative subtypes for patients younger than 50 years. ROC, receiver operating characteristic; AUC, area under the ROC curve; DCNN, deep convolutional neural network.

As shown in **Table 4**, we explored the effectiveness of our DCNN models in different age groups (≤50 and >50 years). In the main cohort, our model had a good performance for distinguishing luminal A from non-luminal A subtypes in the >50 years age group, with the highest AUC of 0.776 (95% CI, 0.649–0.885) and ACC of 83.3% (**Figure 3**). Their SEN and SPC were 89.0% and 65.6%, respectively. In the external testing cohort, our DCNN model achieved AUC values of 0.686 (95% CI, 0.567–0.806) for distinguishing luminal A from non-luminal A subtypes in the >50 years age group. As shown in **Table 5**, our DCNN model also achieved an AUC of 0.712 (95% CI, 0.538–0.874) in the ≤50 years age group for distinguishing the non-TN from TN subtypes (**Figure 3**) in the main cohort, while its diagnostic performance could not be evaluated in the external testing cohort where the TN subtype only has one patient.

**Table 4:** Diagnostic performance of deep convolutional neural network model for differentiating luminal A and non-luminal A subtypes based on patient age in the main cohort.

Experiment	Model	AUC (%)	ACC (%)	SEN (%)	SPC (%)	YI (%)
Age ≤ 50	EfficientNet-B0	58.1	46.4	35.7	92.3	28.0
	DenseNet-121	59.6	53.6	48.2	76.9	25.1
	VGGNet-19	57.4	53.6	48.2	76.9	25.1
Age > 50	EfficientNet-B0	75.2	83.3	90.0	62.5	54.6
	DenseNet-121	77.6	83.3	89.0	65.6	54.6
	VGGNet-19	72.7	81.8	88.0	62.5	50.5

AUC, area under the curve; ACC, accuracy; SEN, sensitivity; SPC, specificity; YI, Youden’s index.

**Table 5:** Diagnostic performance of deep convolutional neural network model for differentiating triple-negative and non-triple-negative subtypes based on patient age in the main cohort.

Experiment	Model	AUC (%)	ACC (%)	SEN (%)	SPC (%)	YI (%)
Age ≤ 50	EfficientNet-B2	71.2	58.5	81.8	53.7	35.5
	DenseNet-121	68.5	63.1	81.8	59.3	41.1
	VGGNet-19	63.5	63.1	72.7	61.1	33.8
Age > 50	EfficientNet-B2	50.4	71.9	36.4	79.8	16.2
	DenseNet-121	51.2	33.1	95.5	19.2	14.6
	VGGNet-19	55.1	68.6	40.9	74.7	15.7

AUC, area under the curve; ACC, accuracy; SEN, sensitivity; SPC, specificity; YI, Youden’s index.

### The Effect of Tumor Size on Differentiating Molecular Subtypes

As shown in **Table 6**, we divided the patients into two groups by the long diameters (≤20 and >20 mm). The results showed that our DCNN model had the best performance in distinguishing luminal A from non-luminal A subtypes for long diameters ≤20 mm, with the AUC, ACC, SEN, SPC, and YI of 0.818 (95% CI, 0.726–0.902), 80.4%, 82.5%, 75.0%, and 57.5%, respectively (**Figure 3**).

**Table 6:** Diagnostic performance of deep convolutional neural network model for differentiating luminal A and non-luminal A subtypes based on tumor sizes in the main cohort.

Experiment	Model	AUC (%)	ACC (%)	SEN (%)	SPC (%)	YI (%)
Long diameter ≤ 20 mm	EfficientNet-B0	80.8	75.9	78.8	68.8	47.5
	DenseNet-121	81.8	80.4	82.5	75.0	57.5
	VGGNet-19	77.3	74.1	76.2	68.8	45.0
Long diameter > 20 mm	EfficientNet-B0	32.6	85.4	100	0	0
	DenseNet-121	43.4	21.3	7.9	100	7.9
	VGGNet-19	34.1	25.8	15.8	84.6	0.4

AUC, area under the curve; ACC, accuracy; SEN, sensitivity; SPC, specificity; YI, Youden’s index.

## Discussion

Medical imaging explored the characteristics from cellular to molecular levels and even the microscopic views to identify the different types of breast cancer to aid in clinical practice. Breast cancer with different molecular subtypes displays diverse, subtle, and overlapping imaging features, which makes accurate diagnosis difficult (16, 17). In this study, we involved different DCNN models for the preoperative differentiation of molecular subtypes of breast cancer, and the diagnostic performance was compared in different patient age groups and tumor size groups, which is not covered in previous studies and could provide more complementary information for real-world radiologists on molecular subtype classification (18, 19).

Based on BI-RADS terminology for US, after standard morphological feature classification, we established a BI-RADS lexicon model for molecular subtype diagnosis to compare with our DCNN model. The BI-RADS lexicon model analysis has been widely applied to tumor characterization, cancer recurrence prediction, and detection of breast cancer for fine-needle aspiration cytology (20, 21). It was reported that using the BI-RADS lexicon model reached a similar performance to that of the radiologists or other machine learning methods. It is common knowledge that the descriptor labeling of breast masses in US was subjective and time-consuming, which prevents it from being applied in the clinical setting. Practically, our DCNN model showed great application potential with results superior to those of the BI-RADS lexicon model in all tasks in both the main cohort and external testing cohort.

Our dataset has a considerable sample size, which is leveraged by our DCNN model to fully mine the hierarchical information from breast cancer US images, and thus the DCNN could cope with a variety of tasks including the stratification analysis of the ages and tumor sizes. According to the breast cancer tumor node metastasis (TNM) stage system, the tumor size is a key reference factor for surgical method selection. A small tumor (tumor size ≤20 mm) is difficult to diagnose by US images, because its morphological characteristics are not obvious, and it is easy to be misdiagnosed as a benign nodule (22). Delayed treatment can impose severe psychological and financial burdens on patients. Moreover, the luminal A subtype is more sensitive to endocrine therapies with a good prognosis when diagnosed and treated early. In our study, the DenseNet-121 DCNN model achieved the best performance for differentiating luminal A from non-luminal A subtypes for tumor sizes ≤20 mm with an AUC of 0.818 (95% CI, 0.726–0.902) and ACC of 75.9%, which could be applied to guide the initial treatment for breast cancer patients.

Several studies have shown that the patients with TN breast cancer are younger (<40 years) than those with non-TN subtypes (23). Therefore, on the basis of age stratification, we further explored the classification effectiveness of our model for this particular subtype of breast cancer. Because of its more aggressive nature and poorer prognosis, early diagnosis and treatment of triple-negative breast cancer have always been a focus of medical research. The study conducted by Saha et al. obtained an AUC of 0.654 through a machine learning model on MRI images for distinguishing TN breast cancer from other subtypes (18). Wu et al. retrospectively analyzed US images and clinical data of 140 cases of surgically confirmed breast cancer (divided into TN and non-TN breast cancer groups) (8). The features of US images and color Doppler images were all analyzed by machine learning methods. Among the twelve US and color Doppler image features finally extracted, there were 8 features with statistical differences between the two groups (p < 0.05). The final diagnosis performance achieved an AUC of 0.88 with a SEN of 86.96% and an SPC of 82.91%. Based on the single-mode image of two-dimensional US for feature extraction, our results achieved a moderate result with the best AUC of 0.712 (95% CI, 0.538–0.874) for differentiating TN from non-TN subtypes among patients with age ≤ 50 years. Hence, the results of this study are crucial for assisting in the diagnosis of TN breast cancer to the maximum extent for younger women. In addition, for the classification of luminal A from non-luminal A subtypes, our model showed a diagnostic AUC of 0.776 (95% CI, 0.649–0.885) and an ACC of 83.3% among patients > 50 years old. This group also performed fairly well in the external testing cohort with an AUC of 0.686 (95% CI, 0.567–0.806). Therefore, for older patients with a high incidence of breast cancer, referring to the diagnosis of our model not only can improve the diagnosis confidence of malignant tumors but also can further obtain the subtype information of classification, which improve patient satisfaction.

Overall, our DCNN models performed moderately in stratified studies based on ages and nodule sizes. This could be attributed to the large variety of image acquisition machines in this study and the single modality of US images. However, from another point of view, it also shows the good applicability of our model to different machines. Due to the small amount of data, the external testing results of the subgroups of our DCNN model were unstable. In the subsequent experiments, we will try to add multi-modal information based on larger sample size for exploration, and the diagnostic performance is expected to be further improved.

There are several limitations to this study. Firstly, compared with other imaging examinations, in the application research of artificial intelligence technology, the high noise, low resolution, non-uniform image standard, and other deficiencies have seriously affected its performance on this tough task of differentiating breast cancer molecular subtypes. Secondly, based on the retrospective collection of two-dimensional US image datasets, there are still deficiencies of inconsistent image quality and limited sample size in this study.

In conclusion, our study has demonstrated that the DCNN model based on US images has the potential to provide a non-invasive method to preoperatively predict the breast cancer molecular subtypes, which could be a reference for clinical treatment arrangement. Specifically, our model could be more valuable in the classification of luminal A from non-luminal A subtypes for patients older than 50 years or whose tumor sizes ≤20 mm. For young patients ≤50 years, our model could be helpful in detecting triple-negative breast cancer more accurately. Future work on expanding the sample size and imaging modalities could further optimize our model and eventually assist radiologists and oncologists in real-world clinical practice.

## Data Availability Statement

The data analyzed in this study is subject to the following licenses/restrictions: The dataset is private and involves patient privacy. Requests to access these datasets should be directed to Rong Wu, https://wurong7111@163.com.

## Author Contributions

CL, SS and JC collected the images and cilinical data. QZ and RW designed the study. HH and YC performed the image processing and the statistical analysis. CL, HH and QZ contributed to the manuscript drafting and editing. All authors approved the manuscript.

## Funding

This work was supported by the National Natural Science Foundation of China (Grants No. 82071931, 82130057, 62071285), program for Shanghai Outstanding Medical Academic Leaders (2019LJ18), the interdisciplinary program of Shanghai Jiaotong university (ZH2018ZDA17), the program from Science and Technology Commission of Shanghai Municipality (No. 20Y11912400), and the 2019 clinical research innovation team of Shanghai General Hospital (No. CTCCR-2019B05).

## Conflict of Interest

The authors declare that the research was conducted in the absence of any commercial or financial relationships that could be construed as a potential conflict of interest.

## Publisher’s Note

All claims expressed in this article are solely those of the authors and do not necessarily represent those of their affiliated organizations, or those of the publisher, the editors and the reviewers. Any product that may be evaluated in this article, or claim that may be made by its manufacturer, is not guaranteed or endorsed by the publisher.
